# p27^Kip1 ^is expressed in proliferating cells in its form phosphorylated on threonine 187

**DOI:** 10.1186/1472-6890-5-3

**Published:** 2005-02-23

**Authors:** Giancarlo Troncone, Juan C Martinez, Antonino Iaccarino, Pio Zeppa, Alessia Caleo, Maria Russo, Ilenia Migliaccio, Maria L Motti, Daniela Califano, Emiliano A Palmieri, Lucio Palombini

**Affiliations:** 1 Dipartimento di Scienze Biomorfologiche e Funzionali, Facoltà di Medicina e Chirurgia, Università Federico II, Napoli, Italy; 2 Istituto Oftalmico, Hospital Universitario Gregorio Maranon, Madrid, Spain; 3 Dipartimento di Biologia e Patologia Cellulare e Molecolare "L Califano", Facoltà di Medicina e Chirurgia, Università Federico II, Napoli, Italy; 4 Dipartimento di Oncologia Sperimentale, Fondazione G. Pascale, Napoli, Italy

## Abstract

**Background:**

G1/S cell cycle progression requires p27^Kip1 ^(p27) proteolysis, which is triggered by its phosphorylation on threonine (Thr) 187. Since its levels are abundant in quiescent and scarce in cycling cells, p27 is an approved marker for quiescent cells, extensively used in histopathology and cancer research.

**Methods:**

However here we showed that by using a specific phosphorylation site (pThr187) antibody, p27 is detectable also in proliferative compartments of normal, dysplastic and neoplastic tissues.

**Results:**

In fact, whereas un-phosphorylated p27 and MIB-1 showed a significant inverse correlation (Spearman R = -0.55; p < 0,001), pThr187-p27 was positively and significantly correlated with MIB-1 expression (Spearman R = 0.88; p < 0,001). Thus proliferating cells only stain for pThr187-p27, whereas they are un-reactive with the regular p27 antibodies. However increasing the sensitivity of the immunocytochemistry (ICH) by the use of an ultra sensitive detection system based on tiramide signal amplification, simultaneous expression and colocalisation of both forms of p27 was shown in proliferating compartments nuclei by double immunofluorescence and laser scanning confocal microscopy studies.

**Conclusion:**

Overall, our data suggest that p27 expression also occurs in proliferating cells compartments and the combined use of both regular and phospho- p27 antibodies is suggested.

## Background

Immunocytochemistry (ICH) is an important method for identification of proteins in cells and in tissues. Since the biological activity of many proteins is dependent on their phosphorylation status, a challenge for immunocytochemistry is to characterize the protein form and not just the total amount [1]. p27^Kip1 ^(p27) is a key inhibitor of cell division that protects tissues from excessive cell proliferation [2]. As a consequence of an altered balance between synthesis and degradation, the amounts of this protein are abnormally low in advanced and poorly differentiated neoplasms [3]. Since p27 expression is readily assessed by ICH, this protein is a prognostic marker quite popular in histopathology [4]. However, little is known on its *in vivo *regulation. p27 cellular levels, copious in quiescent cells undergoing terminal differentiation, are scanty in cycling cells [2]; in these cells p27 is phosphorylated on Thr 187 by cyclin-dependent kinase (cdk) 2 in late G1 [5]. This event leads to enhanced ubiquitination and p27 proteolysis by the proteasome, which marks the restriction point and promotes cell proliferation [5].

Therefore un-phosphorylated ("plain") p27 is representative of the total protein amounts only in quiescent cells, whereas in cycling cells a fraction of p27 is transiently present in the pThr187 form before degradation [5]. Recently, Montagnoli *et al *raised an antibody (Ab), specific for pThr187-p27 that was reactive in immunoprecipitates from proliferating cells and negative in quiescent cells [6]. Even more recently this Ab was shown to be reactive also on paraffin sections [7,8]. The present study was undertaken to assess pThr187-p27 Ab staining pattern in a wide range of normal, dysplastic and neoplastic tissues; its expression was correlated to those of MIB-1, a standard marker of proliferation, and of "plain" p27. The relationship between the two forms of p27 was also studied at a sub cellular level by double immunofluorescence (IF) and laser scanning confocal microscopy (LSCM). Here we show that p27 expression is not restricted to quiescent cells but that it also occurs in proliferating cellular compartments, where it is detectable by regular ICH only in its pThr187 form. Therefore, to fully assess p27 tissue expression both antibodies should be used.

## Methods

### Antibodies

pThr187-p27 was detected by the 71–7100 polyclonal antibody (PcAb) (Zymed Laboratories, San Francisco, CA, USA) and by the sc-16324 PcAb (Santa Cruz Biotechnology Inc., Santa Cruz, CA, USA). These Ab's were raised against a short peptide corresponding to the portion of human p27 containing phosphorylated Thr-187, in order to detect only phospho-p27 and to be unreactive with "plain"-p27. The 71–7100 Ab was previously employed in immuno-precipitation experiments by Western blot, [6,9,10] and to immunostain neoplastic and degenerative human tissue [7,8].

"Plain" p27 protein levels were detected with the K10125 monoclonal Ab (McAb) from (Transduction Laboratories, Lexington, Ky, USA), and with the rabbit PcAb (C-19) (Santa Cruz Biotechnology). These antibodies were previously shown to share the same staining pattern [11]. MIB-1 McAb from Novocastra (Newcastle upon Tyne, UK) was used to stain proliferating cells and as control of antigenic preservation and of successful antigenic retrieval [12].

### Tissues

A wide range of normal, dysplastic and neoplastic tissues was obtained from surgical specimens. At least five samples from each type of normal tissue were processed (Table 1). Dysplastic diseases included five cases of colonic adenoma, five cases of low- and five cases of high grade- squamous intraepithelial lesions (SIL) of the uterine cervix. Several carcinomas were also evaluated, including different tumour types in which p27 down regulation had previously been described, such as invasive squamous cell carcinoma (ISCC) of the oral cavity (n = 7) [13], of the lung (n = 10) [14] and of the uterine cervix (n = 9) [15]; ductal cell carcinoma of the breast (n = 12) [16]; invasive adenocarcinoma of the colon (n = 6) [17] and of the prostate (n = 5) [18]; papillary (n = 6) and anaplastic (n = 5) thyroid carcinoma, [19], glioblastomas (n = 5) [20], and choriocarcinoma (n = 2) [21].

**Table 1:** pThr187-p27, "plain"-p27 and MIB-1 expression in normal tissues.

Tissue type	Phospho-p27	"plain"-p27	MIB-1
**Normal squamous epithelium **skin, tonsil, larynx, cervix.			
*Parabasal layer*	+++	-	++++
*intermediate*	-	++++	+
*Granular layer*	-	+++	-
***Germinal centers ***Tonsil, lymphnode, apendix			
*Mantle cells*	-	++++	-
*Centroblasts*	+++	-	++++
*Centrocytes*	-	+++	-
***Bowel ***Crypt cells			
*Deep*	+++	-	++++
*superficial*	-	++++	-
**Placenta**			
*trophoblast*	+++	-	++++
*syncytiotrofoblast*	-	++++	-
***Kidney***	+/-	+++	+/-
***Lung***	+/-	+++	+/-
**Thyroid**	+/-	+++	+/-
***Prostate***	+/-	+++	+/-

Key: ++++ >80% of positive cells.

+++ 50–80% of positive cells

++ 10–50% of positive cells

+ <10% of positive cells

+/- Only occasional staining

- Negative staining

### Immunostaining techniques

Xylene dewaxed and alcohol rehydrated paraffin sections were placed in Coplin jars filled with a 0.01 M tri-sodium citrate solution, and heated for 3 minutes in a conventional pressure cooker [15]. After heating, slides were thoroughly rinsed in cool running water for 5 minutes. They were then washed in Tris-Buffered Saline (TBS) ph 7.4 before incubating overnight with the specific Ab, diluted as follows: anti-pThr187-p27 (Zymeed) 1:1000; anti-pThr187-p27 (Santa Cruz) 1:200; anti-p27 (Transduction) 1:4000; anti-p27 (Santa Cruz) 1:50; anti-Ki-67 1:50. After incubation with the primary Ab, tissue sections were covered with biotinylated anti-mouse or anti-rabbit immunoglobulins, followed by peroxidase labelled streptavidine (LSAB-DAKO); the signal was developed by using diaminobencidine (DAB) chromogen as substrate.

### Immunostaining controls

pThr187-p27 antibody specificity was controlled by Western blot analysis of MDA MB 468 (breast cancer) and NPA (thyroid papillary cancer) cell lines. These were lysed (50 mmol/L Tris-HCl, pH 7.4, 150 mmol/L NaCl, 0.1% Triton X-100, 5 mmol/L ethylenediaminetetraacetic acid, 1 mmol/L Na_3_VO_4_, and 1 mmol/L phenyl methyl sulfonyl fluoride and protease inhibitors) and 20 to 30 μg of proteins were electrophoresed in sodium dodecyl sulfate-polyacrylamide gel electrophoresis gel and transferred onto nitrocellulose membranes. The membranes were first blocked and then incubated with the primary antibody as it follows: anti-pThr187-p27 (Zymeed), 1:2000 and anti "plain" p27 (Transduction), 1:3000 for 1 hour at room temperature. To confirm equal loading, membranes were immunoblotted with monoclonal anti b-tubulin antibody (1:1000, Santa Cruz). After three washes, filters were incubated with horseradish peroxidase-conjugated goat anti-mouse or anti-rabbit antibodies (1:2000; Amersham, Arlington Heights, IL, USA) for 1 hour at room temperature. Detection of immunocomplexes was performed with an enhanced chemiluminescence system (ECL, Amersham).

pThr187-p27 immunostaining specificity was assessed by several control experiments performed in parallel, in which the primary Ab was either replaced by a similarly diluted normal rabbit serum, or adsorbed with increasing concentrations of its phospho and dephosphopeptides (up to 0.14 mg mL^-1^).

### Quantitative study and statistical analysis

In the neoplastic cases examined, labelling indices for pThr187-p27, "plain" p27 and Ki67/MIB-1 were determined. Adjacent sections were used and counting was performed in similar areas; quantitative analysis performed with a computerised analyser system (Ibas 2000, Kontron, Zeiss) was used to score the nuclei of individual cells for expression of these proteins. As already described, nuclear boundary optical density and Ab threshold were adjusted for each case examined [15]. A minimal threshold was established by counting at least 1000 cells per sample and the results were expressed as a percentage of the total cell population. Statistical analysis was performed by means of SPSS Inc. package. The range of expression of pThr187-p27, "plain" p27 and Ki67/MIB-1 for each neoplastic type is reported in table 2. The relationships among these were analyzed by calculating the nonparametric Spearman R coefficient.

**Table 2:** Range of positive cells for pThr187-p27, "plain"-p27 and MIB-1 expression in different type of carcinomas

**Histotype**	**pThr187-p27**	**"plain" p27**	**MIB-1**
ISCC cervix n = 9	45–60	5–30	50–78
ISCC oral n = 7	40–70	5–15	35–80
ISCC lung n = 10	15–60	5–70	30–70
Thyroid, papillary carcinoma n = 6	1–3	5–60	2–4
Thyroid, anaplastic carcinoma n = 5	23–45	5–11	41–55
Breast carcinoma n = 12	3–55	5–70	5–80
Colonic carcinoma n = 6	30–45	5–80	45–80
Prostate carcinoma n = 5	2.5–10	30–75	4–15
Glioblastoma n = 5	30–50	10–27	50–63
Chorioncarcinoma n = 2	45–55	3–7	60–80

### Double Immunofluorscence (IF) staining and Laser Scanning Confocal Microscopy (LSCM)

Tissue sections from both normal and neoplastic specimens were also stained using double IF labelling for pThr187-p27 and "plain" p27, according to previous studies with minor modifications [22]. The primary anti pThr187-p27 (Zymeed) was incubated (1/1000) for 1 hour, followed by incubation with swine anti rabbit HRP (1/200) (Dako) and Cy3 tyramide amplification (Perkin Elmer Life Sciences). To avoid cross-reactivity due to residual HRP, sections were incubated with 0.3% H_2_O_2 _for 1 hour. The primary mouse McAb anti p27 (1/4000) was detected by overnight incubation at 4°C, followed by goat anti mouse HRP (1/200, DAKO). After washing in TBS, sections were incubated with fluorescein isotiocyanate (FITC) tyramide amplification (Perkin Elmer Life Sciences) and mounted with Vectashield-DAPI mounting medium (Vector). Control sections in which the second primary Ab was omitted were included to ascertain destruction of peroxidase activity. Single IF stained sections as well as colorimetric immunostaining were used as controls of signal specificity. Slides were examined with a Leica TCS SP2 UltraSpectral LaserScan Confocal microscope. FITC was excited at 488 and detected with a bandpass 500 to 550 nm. Cy3 was excited at 514 nm and detected with a bandpass 580 to 655 nm. Series of images were processed with the Leica confocal soft package. Confocal images were captured and imported into Adobe Photoshop 7 (Adobe Systems, Mountain View, CA) and processed with an eMac personal computer.

## Results

### pThr187-p27 expression in normal tissues

**Figure 1:**
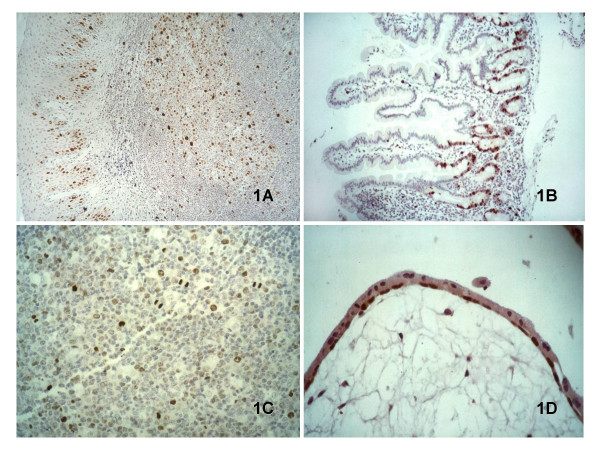
p27 expression was detected by the pThr187 Ab p27 only in the proliferative compartments of normal tissues. In tonsil (A) both parabasal squamous cells and lymphoid germinal cells were stained. In intestinal epitehlium only the nuclei of deep crypt cells were stained (B). Within the germinal centres, the outer rim of centroblasts and mitotic cells were strongly positive for pThr187-p27, whereas centrocytes were less labelled (C). Trophoblastic villi of placenta show pThr187-p27 intense staining only in the cytotrophoblastic layer and not in the syncytiotrophoblastic layer (D).

Similar results were obtained with both anti pThr187-p27 Ab's, with only proliferating tissue compartments being stained. This pattern overlapped to that of Ki67/MIB-1, whereas it was different to that shown by both Mc and Pc Ab's directed to "plain" p27 (Table 1). Stratified squamous epithelium showed the same staining pattern in skin, tonsil, larynx and uterine cervix. In analogy to the Ki67/MIB-1 staining, pThr187-p27 was expressed by parabasal cells (Figure 1A); on the contrary, nuclei of the more superficial layers showed intense "plain" p27 nuclear labelling, as already described [13,15]. In small intestine and colon (Figure 1B), in analogy to MIB-1, pThr187-p27 showed positivity in nuclei of deep crypt cells. On the contrary, the upper half of the crypts was stained by "plain" p27 [17]. In germinal centres of normal lymph nodes, tonsil and appendix the expression of the phosphorylated form of p27 was clearly detectable (Figures 1A and 1C). Within the germinal centres, pThr187-p27 showed a signal distribution similar to that of the Ki-67 protein; the outer rim of centroblast and mitotic cells were strongly positive, whereas centrocytes were less frequently labelled. This pattern of expression was opposite to the one identified for "plain" p27: negative staining for centroblasts and strong staining in most centrocytes, mantle cells and interfollicular small lymphocytes [11]. Trophoblastic villi of placenta showed pThr187-p27 intense staining only in the cytotrophoblastic layer and not in the syncytiotrophoblastic layer (Figure 1D). On the contrary "plain" p27 showed intense staining only in the syncytiotrophoblast overlying the villus, as already described [21].

In other normal tissues, comprising breast, lung, kidney, pancreas, prostate, thyroid and parathyroid, "plain" p27 was expressed by the vast majority of cells, whereas pospho-p27 single labelled cells could be made out by meticulous scrutiny; in these tissues also MIB-1 staining was sporadic.

### pThr187-p27 expression in dysplastic and neoplastic tissues

**Figure 2:**
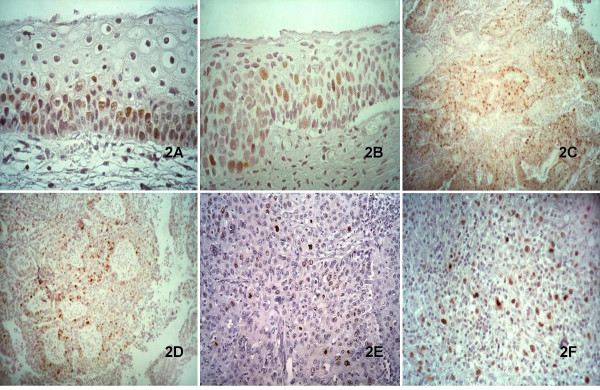
The relationship between phospho-p27 expression and proliferation was evident both in low- (A) and high- (B) grade SIL, with its expression correlating well with the extent of the dysplastic cell population. Intense staining for phospho-p27 was also observed in the neoplastic cells of colonic (C) and lung (D) adenocarcinoma, in cervical squamous invasive carcinoma (E) and in choriocarcinoma (F)

As examples of dysplastic lesions, the expression of the pThr187-p27 was assessed in low-and in high grade squamous intraepithelial lesions (SIL) of the uterine cervix and in colonic adenoma. According to the relationship between phospho-p27 expression and proliferation, in low grade SIL the dysplastic basal and parabasal cells were in most instances positive for the expression of both pThr187-p27 (Figure 2A), and Ki-67; these were instead negative for "plain" p27 whose staining was confined to the intermediate and superficial squamous cells, as described [15]. In high grade SIL, the expression of phospho-p27 correlated well with the extent of the dysplastic cell population (Figure 2B), whereas "plain" p27 reactivity was restricted to very superficial layers displaying squamous differentiation. In colonic adenoma pThr187-p27 positive cells were randomly located throughout the crypts and in contrast to that seen in normal mucosa, also superficial cells were stained.

A wide range of different tumours was examined, in order to assess whether staining for phospho-p27 may yield diagnostic information in addition to those provided by "plain p27" (Table 2). As a general rule, the expression of the two forms of p27 was alternative and dependent on the degree of tumour differentiation, recapitulating the pattern featured by normal and dysplastic tissues; phospho-p27 strongly labelled aggressive tumours, whereas "plain p27" staining was only retained by well differentiated tumours. Thus, poorly differentiated and highly proliferating adenocarcinoma of the breast, colon and prostate were strongly labelled by the pThr187-p27 Ab, (Figure 2C–D) whereas "plain p27" staining was only retained by well differentiated neoplasms. The above staining differences between the two forms of p27 were also evident in squamous cell carcinomas. Poorly differentiated neoplasms, composed of nests of small undifferentiated cells with high MIB-1 index and minimal keratinisation, showed intense staining for pThr187-p27 (Figure 2E) whereas "plain p27" labelled those neoplasms showing abundant keratin, squamous pearl formation and low mitotic activity. Similarly, anaplastic thyroid carcinoma showed pThr187-p27 staining stronger than papillary carcinomas, while in this latter "plain p27" was prevalent (Table 2). An intense pThr187-p27 expression was also found in choriocarcinoma (Figure 2F), in which the level of "plain-p27" staining was instead low. The Spearman's correlation coefficient for continuous variables revealed a positive and a significant correlation between pThr187-p27 staining and MIB-1 expression (Spearman R = 0.88; p < 0,001). On the contrary both pThr187-p27 and "plain" p27 (Spearman R = -0.61; p < 0,001) and MIB1 and "plain" p27 (Spearman R = -0.55; p < 0,001) showed significant inverse correlations.

### Immunostaining controls

**Figure 3:**
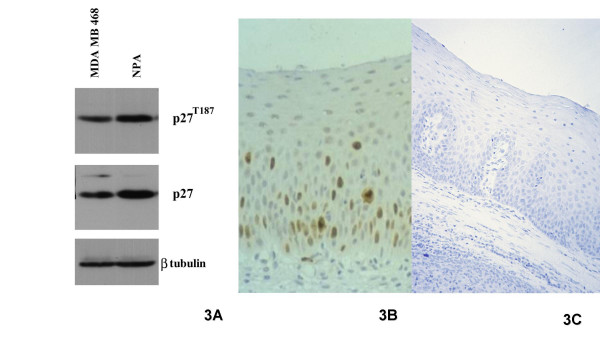
Western blot analysis of MDA MB 468 (breast cancer) and NPA (thyroid papillary cancer) cell lines revealed a single anti-pThr187-p27 band, corresponding to that shown by the regular p27 antibodies. (A). pThr187 staining of parabasal squamous cells (B) abolished by absorption of the antibody with the immunizing peptide.

Western blot analysis of MDA MB 468 (breast cancer) and NPA (thyroid papillary cancer) cell lines revealed a single anti-pThr187-p27 band, whose molecular weight (27 kDa) corresponded to that showed by "plain"-p27 (Fig. 3A). pThr187-p27 immunostaining specificity was confirmed by: (i) the disappearance of the signal when the primary Ab was replaced by a similarly diluted normal rabbit serum; (ii) the progressive signal quenching due to the competitive inhibition between the increasing concentrations of the phosphopeptide and the phosho-p27 Ab. (Fig. 3 B–C).

#### Double IF & LSCM studies

**Figure 4:**
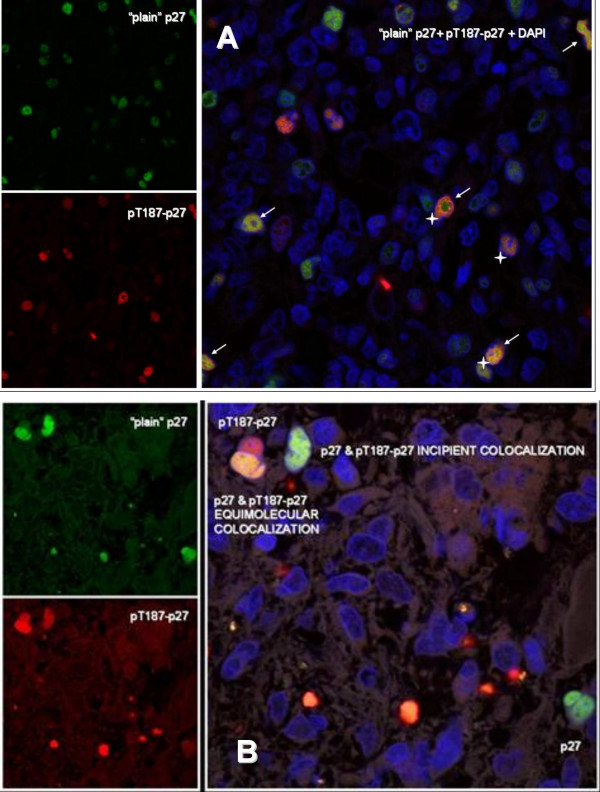
Coexpression of the forms of p27 in lymphoid germinal center (A) and in glioblastomas (B). Simulataneous detection of p27 by both regular (green) and pThr187 (red) antibodies. Signal generated by the two antibodies Yellow areas (arrows) showed the colocalitation of the signal generated by both antibodies.

Tissue sections from both normal and neoplastic specimens were also stained using double IF labelling for pThr187-p27 and "plain" p27 and analyzed by LSCM. The use of an ultra sensitive detection system based on tiramide signal amplification for IF staining, revealed a more precise pattern of protein expression and distribution, slightly different to results above shown of regular colorimetric IHC. In proliferative compartments of normal tissues the cells frequently showed simultaneous expression of the two forms of p27; in these cells, pThr187-p27 was more localized as an inner rim along the nuclei membrane, whereas "plain" p27 was more centrally located in the same nuclei with an intermediate area of colocalization (Fig. 4A). Similarly neoplastic nuclei also showed simultaneous expression of the two p27 forms, frequently displaying high amounts of pThr187-p27 throughout the whole nucleoplasm, colocalizing with "plain" p27. (figure 4B).

## Discussion

p27 is an approved marker for quiescent cells, extensively used in histopathology and cancer research. However here we showed that an unusual p27 expression pattern may be obtained when only its portion phosphorylated on threonine 187 is stained. By using a specific phosphorylation site antibody, p27 expression may be detected in the proliferative compartments of squamous and intestinal epithelia, of lymph node germinal centres, of trophoblastic villi, of cervical and colonic pre-invasive lesions and of several carcinomas types. These results are similar to those reported *in vitro *by Montagnoli *et al*, who also detected p27 expression in proliferating cells by the pThr187-p27 Ab [6]. The same antibody, here, employed to stain a wide range of normal, dysplastic and neoplastic tissues yielded a pattern similar to that of Ki-67 (Spearman R = 0.88; p < 0,001), underlining the close association between cell proliferation and the expression of that portion of p27 targeted for degradation. Since pThr187-p27 Ab should react with cells from late G1 through G2-M, whereas MIB-1 stains throughout the entire cell cycle, it is also conceivable that the pThr187-p27 Ab stained a lower percentage of cells than MIB-1/Ki-67 in both normal (Table 1) and neoplastic tissue. (Table 2)

In this study we showed that p27 expression does not feature only one pattern, but that two different staining may be obtained by the use of either regular or pThr187 antibodies; in fact, when the overall data relative to the expression of p27 by both antibodies in tumours were analysed, a significant inverse correlation between the two forms was found (Spearman R = -0.61; p < 0,001). This data reflects the different relationship between each form of p27 and tissue differentiation:aggressive tumours were strongly labelled by phospho-p27 Ab, whereas plain-p27 was prevalent in well differentiated tumours. Thus the ratio between the two forms of p27, more than the total protein amounts, could yield information on tumour differentiation and behaviour.

Since cdk2 activity is low in differentiating cells and p27 is only present in its un-phosphorylated form, it is reasonable that squamous superficial layers, upper half of the intestinal crypts, lymphoid mantle cells and syncytiotrophoblastic cells only react with the regular antibodies. More intriguing is the search for the reasons explaining why in proliferating cells p27 is detected by the pThr187-Ab and missed by the regular antibodies. The possibility of an aberrant cross-reactivity by the pThr187-Ab was excluded both by Western blot analysis and by phosphopeptide adsorbtion. Montagnoli *et al*, who also confirmed antibody specificity by biochemical assays, showed that p27 expression may be better detected by using both antibodies than either alone and that there is a portion of p27 that is only recognizable by the anti-phospho Ab [6]. Thus, in proliferating cells anti-pThr187 Ab may be more efficient than the regular p27 antibodies, being protein levels low and mainly present in their phosphorylated form. To test this hypothesis, the sensitivity of the p27 detection by the regular antibodies was increased by the use of an ultra sensitive detection system based on tiramide signal amplification and observed at high resolution by laser scanning confocal microscopy. The results obtained by this approach were slightly different to those obtained by colorimetric IHC, as p27 was simultaneously detected by both antibodies in the same nuclei of the proliferating compartments; in particular the signal generated by both antibodies showed specific colocalitation by double immunofluorescence.

## Conclusion

In this study we have reported the following observations: (1) pThr187-p27 Ab selectively stains proliferating cells; (2) this staining does not identify the "plain" p27 protein, expressed by quiescent cells. Therefore, the pThr187-p27 antibody is a useful tool to study p27 in vivo regulation. Indeed, the pattern of expression observed in this study, strongly suggest that the anti-pThr187-p27 Ab identifies proliferating cells. Considering the importance of the different biological functions of p27: regulator of cell growth, cell differentiation, contact inhibition, apoptosis, protection against immunological aggression, and protection against environmental stress, among others, the combined use of both "plain" and pThr187 p27 antibodies will be a useful tool to better characterize the biology of these conditions.

## Competing interests

The author(s) declare that they have no competing interests.

## Authors' contributions

GT conceived the study and wrote the manuscript; JCM carried out the LSCM study; AC scored the immunostaining; AI coordinated the study; MR performed the peptide experiments; PZ evaluated the tumour pathological features; IM participated in its design and coordination and helped to draft the manuscript; MLM carried out the double immunostainings; DC carried out the Western blot analysis; EAP performed the statistical analysis. LP participated in the design of the study and evaluated the results.

All authors read and approved the final manuscript

## Pre-publication history

The pre-publication history for this paper can be accessed here:


